# Accuracy and Documentation of Richmond Agitation‐Sedation Scale Assessments in a Cardiothoracic Critical Care Unit: A Prospective, Cross‐Sectional Audit

**DOI:** 10.1111/nicc.70531

**Published:** 2026-06-16

**Authors:** Lajos Szentgyorgyi, James Alder, Cara Godfrey, Samuel Henry Howitt, Heather Iles‐Smith, Bhuvaneswari Krishnamoorthy

**Affiliations:** ^1^ School of Health and Society The University of Salford Salford UK; ^2^ Wythenshawe Hospital, Cardiothoracic Critical Care Unit Manchester University NHS Foundation Trust Manchester UK; ^3^ Faculty of Biology, Medicine and Health The University of Manchester Manchester UK; ^4^ Division of Cardiovascular Sciences The University of Manchester Manchester UK; ^5^ Northern Care Alliance NHS Foundation Trust, Centre for Clinical and Care Research Salford UK

**Keywords:** critical care, nursing, Richmond Agitation‐Sedation Scale (RASS), sedation audit

## Abstract

**Background:**

Sedation is a fundamental component of critical care and requires regular, accurate assessment to support its safe titration and minimise harm. International guidelines and local policy at the Wythenshawe Hospital's Cardiothoracic Critical Care Unit mandate hourly documentation of the Richmond Agitation‐Sedation Scale (RASS).

**Aims:**

This audit evaluated adherence to these standards and explored factors associated with documentation completeness and assessment accuracy.

**Study Design:**

A prospective, cross‐sectional audit of bedside RASS assessment was conducted without prior notification. Nursing RASS scores were compared with expert auditor ratings. Data were collected using an anonymous, secure, auditor‐completed online survey. Statistical analysis examined the relationship between documentation completion and RASS accuracy.

**Results:**

Seventy‐six nursing assessments were included. Most nurses were Band 5, with a median of 3.5 years of experience. Patients were predominantly male, mechanically ventilated and receiving sedation. Overall, 75% of nurse RASS assessments (*n* = 57) were accurate; however, only 33% (*n* = 25) were documented hourly as required by policy. Greater nursing experience was associated with improved accuracy in RASS. Deeper sedation was associated with lower agreement between nurse and expert assessments. Higher self‐reported confidence was associated with poorer completion of documentation. Most discrepancies were small, with nurse and expert ratings differing by one RASS point.

**Conclusions:**

This audit identified important gaps in the accuracy and documentation of nurse‐led RASS assessment in a cardiothoracic critical care setting. The findings suggest that perceived familiarity and confidence may not be sufficient to ensure accurate and consistently documented sedation assessment.

**Relevance to Clinical Practice:**

Suboptimal RASS documentation and scoring accuracy may limit reliable titration of sedation in critically ill patients and reduce the effectiveness of protocolised sedation strategies. These findings support targeted staff education, reinforcement of standardised assessment processes and improvements in documentation systems to strengthen sedation monitoring and patient safety.

## Key Points

- *Documentation of sedation assessments is suboptimal*. Only one‐third of Richmond Agitation–Sedation Scale (RASS) assessments were documented hourly as required, indicating a significant gap between policy and clinical practice.
- *One in four RASS assessments is inaccurate*. A substantial proportion (25%) of nurse‐assessed RASS scores did not match expert ratings, highlighting limitations in the reliability of bedside assessment.
- *Greater clinical experience improves accuracy, but not documentation*. More experienced nurses demonstrated significantly better RASS scoring accuracy, yet experience was not associated with improved documentation compliance.
- *Confidence does not equate to accuracy or compliance*. Higher self‐reported confidence was not associated with improved scoring accuracy and was associated with poorer documentation completeness, suggesting potential overconfidence.
- *Deeper sedation is associated with lower scoring agreement*. Lower (more negative) RASS scores were associated with greater discrepancies between nurse and expert assessments, suggesting particular difficulty in evaluating deeply sedated patients.
- *Incomplete or inaccurate assessments may compromise sedation management*. Suboptimal documentation and scoring accuracy may limit reliable titration of sedation, potentially affecting patient safety and clinical outcomes.
- *Targeted education and system‐level interventions are needed*. Findings support the need for structured staff training, improved documentation systems and ongoing audit cycles to enhance the quality of sedation monitoring.

## 1 Introduction

Sedation and analgosedation, the use of pharmacological analgesic agents to maintain analgesia and sedation, are crucial in the management of patients in intensive care units (ICUs) [1, 2]. These interventions aim to reduce patient anxiety, alleviate stress associated with mechanical ventilation and prevent harm resulting from pain or agitation related to illness or medical procedures [1, 2, 3]. However, inappropriate sedation, whether excessive or insufficient, can lead to significant complications, underscoring the need for precise and reliable sedation monitoring to allow appropriate titration of the medications used [3, 4, 5]. The Pain, Agitation/Sedation, Delirium, Immobility, and Sleep (PADIS) guidelines recommend that nursing staff use validated, objective tools to assess sedation levels regularly [1, 2].

At Manchester University NHS Foundation Trust, Wythenshawe Hospital's Cardiothoracic Critical Care Unit (CTCCU), hourly assessments using the Richmond Agitation‐Sedation Scale (RASS) are mandated to guide nurse‐led sedation management [6]. Although processed electroencephalogram‐based (pEEG) sedation‐monitoring technologies are available in the department, they have not yet been implemented in routine clinical practice. This is partly due to mixed evidence regarding their clinical benefit, challenges in interpretation and the additional training and resources required for effective use [7, 8, 9]. This clinical audit evaluates compliance with two key standards at Wythenshawe Hospital's CTCCU: the frequency of documented RASS assessments in electronic patient records and the accuracy of these assessments. The findings will inform targeted improvements in sedation monitoring practices within this quaternary heart and lung transplant and extracorporeal membrane oxygenation (ECMO) centre.

## 2 Methods

### 2.1 Study Design

This study was a prospective, cross‐sectional audit of bedside RASS scoring, comparing nursing assessments with expert auditor ratings. The manuscript was prepared in accordance with the SQUIRE 2.0 (Standards for QUality Improvement Reporting Excellence) guidelines to ensure transparent and rigorous reporting of this quality improvement initiative [10]. The clinical audit was registered and approved by the clinical audit department on 12 September 2023.

### 2.2 Data Collection

Seventy‐six patients in the CTCCU were selected and assessed between 2 October 2023 and 3 April 2025, using a pragmatic, unannounced audit sampling approach. Patients were included if they were adults, cared for by a bedside nurse in the CTCCU at the time of the audit, and if a RASS assessment was clinically appropriate (e.g., the patients were not paralysed). Patients were excluded when a RASS score was not meaningful or could not be interpreted reliably, such as patients with non‐pharmacological coma, under neuromuscular blockade, severe neurological impairment affecting arousal, profound hearing impairment or language barriers that prevented relevant interaction and situations where approaching the patient or nurse would have disrupted urgent clinical care. Patients receiving intravenous or enteral sedation were eligible, as were non‐sedated patients when RASS assessment remained clinically appropriate.

Patients were not selected from a pre‐generated randomisation list; rather, audits were undertaken intermittently when auditors were available and when data collection would not interfere with clinical care. To reduce selection bias and improve representativeness, the audit team approached different bedside nurses and patients across the CTCCU over an extended period, including different clinical shifts and seasons where feasible. A formal randomisation process would have been impractical due to potential interference with urgent clinical care, and it is not required for an observational audit project. Assessments were conducted without prior notification to minimise staff's preparatory behaviour, reduce observer bias and better reflect routine clinical practice.

Sedative, analgesic or anti‐agitation medication was not changed for the audit, and no such changes occurred between the bedside nurse's RASS assessment and the auditor's independent assessment. Consequently, both assessments reflected the same clinical sedation or agitation state, allowing agreement in RASS scoring to be assessed. The audit did not evaluate sedation titration, medication management or changes in sedation or agitation over time.

The audit team comprised an advanced critical care practitioner and a medical student, both trained by an experienced intensive care physician. Standardisation was supported by the use of the Manchester University NHS Foundation Trust's training video on RASS assessments during auditor training. Data were collected anonymously using a Microsoft Forms survey (Microsoft Corporation, Redmond, WA, USA) and securely stored in an NHS cloud‐based storage system. Data collection incorporated discussions with the bedside nurse, review of electronic patient records and an independent auditor assessment of each patient's RASS score. Patient data collected included age, gender, ventilation status and use of intravenous or enteral sedation. Nurse‐related variables included pay band, years of experience, familiarity with RASS, including its purpose, frequency and methodology, recall of departmental training within the previous 2 years, perceived need for further training and confidence in assessment accuracy, measured using a 5‐point Likert‐type scale from 1 (not confident) to 5 (absolutely confident).

Electronic patient records (Epic Systems, Verona, WI, USA) were used to determine whether the RASS assessment had been recorded hourly, incompletely or not at all during the nursing shift in which the assessing nurse commenced duty. Nurses were asked to provide a simultaneous RASS score followed by an independent assessment conducted by a trained auditor. Assessment accuracy was defined as agreement between the nurse‐recorded RASS score and the trained auditor's RASS score.

### 2.3 Statistical Analysis

Descriptive statistics were used to summarise patient and nurse characteristics as well as the accuracy and completeness of RASS assessments. Non‐normally distributed continuous and ordinal variables were reported as medians and interquartile ranges (IQR). Categorical variables were summarised using frequencies and percentages.

Statistical analyses were performed using Microsoft Excel (Microsoft Corporation, Redmond, WA, USA) and IBM SPSS Statistics 29 (IBM Corp., Armonk, NY, USA). Associations between assessor‐related factors and completeness of RASS documentation and accuracy of nurses' RASS scoring were explored. The Mann–Whitney *U*‐test was used to compare ordinal or continuous variables between two independent groups; the Hodges–Lehmann estimator reported median differences with 95% confidence intervals (CIs). The Kruskal–Wallis test was used to compare three or more groups. Associations between categorical variables were assessed using the chi‐square test of independence, with Fisher's exact test used where appropriate. A *p*‐value ≤ 0.05 was considered statistically significant.

## 3 Results

### 3.1 Demographics

The majority of nurses (89%, *n* = 68) were Band 5, representing the most junior level of registered nursing staff, with a median unit experience of 3.5 (IQR 1.5–5.5) years (Table 1).

**TABLE 1:** Summary of cohort characteristics.

Category			Frequency	Percentage of total
Nursing staff characteristics	Pay band	5	68	89%
		6	8	11%
	Median years of experience (IQR)		3.5 (1.5–5.5)	—
Patient characteristics	Sex	Male	50	66%
		Female	26	34%
	Age (years)	30–39	7	9%
		40–49	8	11%
		50–59	15	20%
		60–69	18	24%
		70–79	20	26%
		80–89	8	11%
		Median (IQR)	64.5 (54.0–75.0)	—
	Intubated and ventilated	Yes	52	68%
		No	24	32%
	Receiving IV sedatives	Yes	48	63%
		No	28	37%
	Receiving enteral sedatives	Yes	36	47%
		No	40	53%
RASS assessment	Familiar with RASS	Yes	65	86%
		No	11	14%
	Received departmental RASS assessment training in the last 2 years	Yes	50	66%
		No	26	34%
	Requiring more RASS assessment training	Yes	19	25%
		No	57	75%
	Confidence with RASS (1 = low, 5 = high)	1	0	0%
		2	4	5%
		3	15	20%
		4	24	32%
		5	33	43%
		Median (IQR)	4 (3.1–4.9)	—
	Documentation of RASS assessment	Complete	25	33%
		Incomplete	29	38%
		Absent	22	29%
	RASS assessment accuracy	Correct	57	75%
		Incorrect	19	25%

Abbreviations: IQR, interquartile range; IV, Intravenous, RASS, Richmond Agitation‐Sedation Scale.

**FIGURE 1:**
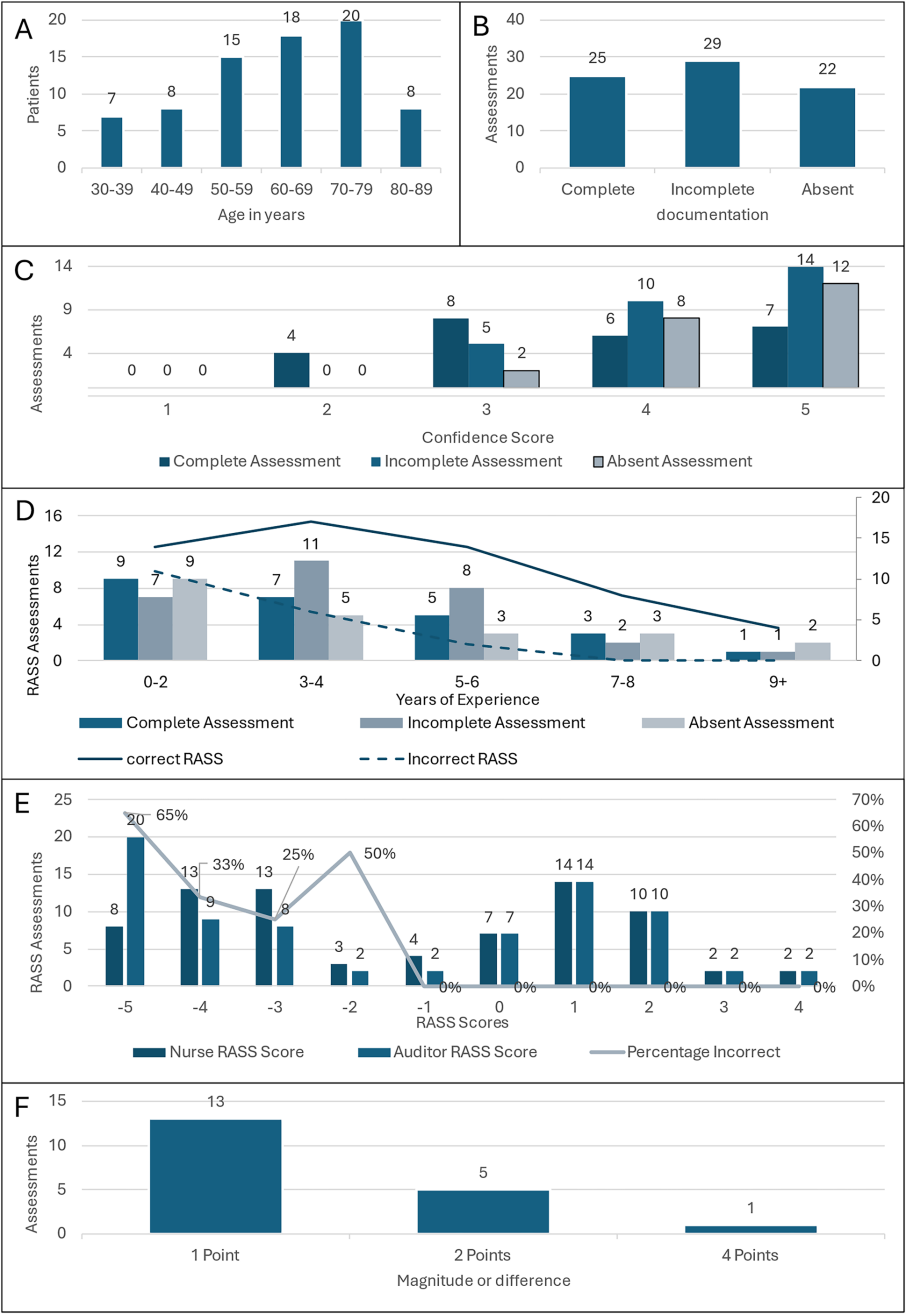
(A) The number of patients by age group. (B) Completion of RASS assessment documentation. (C) Confidence scores by degrees of completion of the RASS assessments (1 [not confident] and 5 [absolutely confident]). (D) Distribution of nurses' RASS assessment documentation by degree of completion, and accuracy of RASS assessments stratified by years of clinical experience. (E) Comparison of nurse‐recorded and auditor‐verified RASS assessments across all RASS score categories, including the proportion of incorrect assessments at each score. (F) Nurse–expert RASS scoring discrepancies: Frequency and magnitude. RASS, Richmond Agitation‐Sedation Scale.

Among patients, 66% (*n* = 50) were male with a median age of 64.5 (IQR 54.0–75.0) years, and the most represented age group was 70–79 years (26%, *n* = 20) (Table 1, Figure 1A). Most patients were intubated and mechanically ventilated (68%, *n* = 52) and received intravenous sedation (63%, *n* = 48), while 47% (*n* = 36) received enteral sedation. Patients could receive intravenous sedation, enteral sedation, both or neither.

Although 86% of nurses reported familiarity with RASS, including its recommended frequency, documentation was fully complete in only 33% of cases (*n* = 25) (Table 1, Figure 1B). While 34% of nurses did not recall receiving departmental RASS training in the past 2 years, only 25% expressed a perceived need for additional training. Despite this, self‐reported confidence in assessment accuracy was high: 43% of nurses (*n* = 33) were ‘absolutely confident’, with a median confidence score of 4 (IQR 3.1–4.9) out of 5. Overall, 75% of nurse assessments (*n* = 57) matched expert assessments.

### 3.2 Associations With RASS Documentation Completion

Table 2 indicates no significant association between nurses' pay band, years of experience or recent training and the completeness of RASS documentation. However, as illustrated in Figure 1C, higher confidence was observed among nurses in groups with less complete documentation (*H* ^2^ = 8.851, *p* = 0.012).

**TABLE 2:** Factors affecting the RASS documentation.

Category		RASS assessment documentation			Statistical results		
		Complete	Incomplete	Absent	*χ* ^2^ (df)	*H* (df)	*p*
Pay band	5	23	26	19	—	—	0.897
	6	2	3	3			
Years of experience	0–2	9	7	9	—	0.187 (2)	0.911
	3–4	7	11	5			
	5–6	5	8	3			
	7–8	3	2	3			
	9+	1	1	2			
	Median (IQR)	3.5 (1.0–5.5)	3.5 (3.5–5.5)	3.5 (1.0–5.5)			
Departmental RASS training in the last 2 years	Yes	17	19	14	0.101 (2)	—	0.951
	No	8	10	8			
Confidence with RASS (1 = low, 5 = high)	1	0	0	0	—	8.851 (2)	**0.012**
	2	4	0	0			
	3	8	5	2			
	4	6	10	8			
	5	7	14	12			
	Median (IQR)	4 (3.0–5.0)	4 (4.0–5.0)	5 (4.0–5.0)			

*Note:* Significant *p*‐values are bolded.

Abbreviations: df, degrees of freedom; H, Kruskal–Wallis H statistic; IQR, interquartile range; IV, intravenous; RASS, Richmond Agitation‐Sedation Scale; *χ* ^2^, chi‐squared statistic.

### 3.3 Associations With RASS Score Accuracy

As shown in Figure 1F, the majority of assessments (75%, *n* = 57) were accurate, and when they differed, 68% of the discrepancies (*n* = 52) between nurse and expert auditor scores were of only one point. However, one discrepancy was four points. Lower RASS scores, as determined by the auditor, were associated with a higher likelihood of incorrect assessments by the nursing staff (U = 127, *p* < 0.001) (Table 3, Figure 1E). All inaccuracies occurred when expert auditor‐assigned RASS scores were < 0, and no inaccurate assessments were observed when the expert auditor‐rated RASS was ≥ 0.

**TABLE 3:** Factors affecting the RASS assessment accuracy.

Category		RASS assessment accuracy		Statistical result			
		Correct	Incorrect	*χ* ^2^ (df)	*U*	Median diff (95% CI)	*p*
Pay band	5	49	19	—	—	—	0.189
	6	8	0				
Years of experience	0–2	14	11	—	251	2.00 (1.00–3.00)	**< 0.001**
	3–4	17	6				
	5–6	14	2				
	7–8	8	0				
	9+	4	0				
	Median (IQR)	3.5 (3.5–5.5)	1 (1.0–1.0)				
Training in the last 2 years	Yes	36	14	0.474 (1)	—	—	0.675
	No	21	5				
Confidence with RASS (1 = low, 5 = high)	1	0	0	—	683	0.00 (−1.00–0.00)	0.070
	2	4	0				
	3	13	2				
	4	18	6				
	5	22	11				
	Median (IQR)	4 (3.0–5.0)	5 (4.0–5.0)				
RASS score	−5	7	13	—	127	—	**< 0.001**
	−4	6	3				
	−3	6	2				
	−2	1	1				
	−1	2	0				
	0	7	0				
	1	14	0				
	2	10	0				
	3	2	0				
	4	2	0				
	Median (IQR)	0 (−3.0 to 2.0)	−5 (−5.0 to −5.0)				

*Note:* Significant *p*‐values are bolded.

Abbreviations: CI, confidence intervals; df, degrees of freedom; diff, difference; IQR, interquartile range; IV, intravenous; RASS, Richmond Agitation‐Sedation Scale; *U*, Mann–Whitney *U*‐test statistic; *χ* ^2^, chi‐squared statistic.

Neither training nor self‐reported confidence was associated with the accuracy of RASS assessments (Table 3). Although no Band 6 nurses recorded an incorrect assessment (*n* = 8), the small sample size limits interpretation, and no statistically significant association was found between pay band and accuracy (*p* = 0.189). Years of experience in the CTCCU were significantly associated with improved accuracy (U = 251, *p* < 0.001), with a median difference of 2.00 years between those performing correct and incorrect assessments (95% CI: 1.00–3.00) (Figure 1D).

## 4 Discussion

### 4.1 Associations With RASS Documentation Completion

The findings demonstrate that RASS documentation completion remains below the expected standard for hourly recordings (Figure 1B, Table 1). This may reflect factors such as high nursing workload and the relatively low prioritisation of sedation scoring. In intensive care settings, time‐motion studies suggest that documentation can account for up to one‐third of nurses' working time [11], potentially detracting from direct patient care [12]. This competes with time‐sensitive monitoring tasks and sedation management [12]. However, workload and task prioritisation were not directly measured in this study.

Although 86% of respondents reported familiarity with recommended assessment frequency, incomplete documentation suggests a potential gap between knowledge and practice. Educational interventions may help address this discrepancy. Especially because less experienced nurses were primarily responsible for routine patient assessments. Ramoo et al. demonstrated that combined theoretical and practical training enhanced critical care nurses' knowledge, scoring accuracy and competence in sedation management. Furthermore, they also identified that workload remained a key perceived barrier, indicating that education alone may be insufficient to address systemic constraints [13, 14]. As bedside documentation was used as a proxy for assessment completion, these findings should be interpreted cautiously, as assessments may have been performed but not recorded.

Confidence was negatively associated with documentation completion, with higher confidence observed among nurses with less complete documentation (Figure 1C). Notably, confidence level was not associated with assessment accuracy. Yang et al. found that research on clinical judgement in nursing reveals systematic miscalibration and overconfidence that intensify with decision difficulty, and that comparable overconfidence has been documented in critical care nursing [15, 16].

### 4.2 Associations With RASS Score Accuracy

Greater nursing experience was associated with RASS scoring accuracy, with a median difference of 2 years between correct and incorrect assessments. However, experience was not associated with improved documentation completion, as no significant relationship was found between experience and documentation rates (Figure 1D), suggesting that accuracy and compliance represent distinct domains of performance. Potential auditor bias should also be considered, as the auditor scores were obtained immediately after the auditor observed the nursing assessment. However, the original adult RASS validation study reported high inter‐rater reliability between a nurse educator and 27 RASS‐trained bedside nurses, supporting the view that, when those measuring the score are trained and the instrument is used as intended, agreement across bedside assessors can be high [6].

Lower (more negative) RASS scores were associated with reduced agreement between nurse and auditor assessments (Figure 1E), highlighting the inherent challenges in accurately assessing patients under deep sedation [1, 2, 17]. Despite this, most scoring discrepancies were minor (Figure 1F), and importantly, all inaccuracies occurred at negative RASS scores, with no errors observed at scores ≥ 0. Notably, the current audit found that nurses were recording RASS scores without systematically assessing patients' responses to verbal or physical stimuli, potentially compromising the validity of the assessments [6, 18]. One possible explanation is that nurses considered a broader temporal clinical impression rather than a point‐in‐time assessment, although this hypothesis requires further investigation. These findings reinforce the importance of adherence to standardised protocols to ensure the reliability and validity of sedation assessments, ultimately improving patient monitoring and outcomes [18, 19, 20, 21, 22].

### 4.3 Clinical Implications of the Findings

Our clinical audit found that 25% of RASS assessments were inaccurate, and only 33% of cases were fully documented during the assessed nursing shifts. These deficiencies may limit the ability to titrate sedation accurately and reliably, thereby decreasing the effectiveness of protocolised sedation strategies. While this study did not evaluate patient outcomes, inappropriate depth of sedation is associated with adverse outcomes [1, 2, 20, 21, 23]. A systematic review and meta‐analyses have identified that deep sedation in mechanically ventilated patients was associated with increased mortality and prolonged hospital stay [23]. Therefore, interventions to assess and target sedation depth early warrant further investigation and may improve patient outcomes [23].

Reliable bedside RASS assessment can be achieved through structured education, robust implementation and a quality assurance policy. For example, Vasilevskis et al. reported substantial agreement between bedside and reference RASS ratings across 6880 paired assessments [24]. Pun et al. demonstrated high compliance and strong interrater agreement following a structured implementation that included staged education and daily monitoring [25]. In contrast, Anderson et al. demonstrated that usual‐care bedside RASS assessments can become poorly concordant with protocolised assessments over time, describing this decline as ‘care erosion’ [26].

### 4.4 Recommendations

1. Targeted staff training
  Focus on junior nurses and those with less experience, and ask them for their preferences on how to improve RASS assessments. Training should emphasise the correct RASS methodology, adherence to protocols and documentation. Incorporating feedback and reflective practice may help address overconfidence.
2. Improved documentation practices
  Implement standardised protocols and electronic prompts within patient records to support consistent hourly documentation.
3. Adjunctive monitoring technologies
  Evaluate the role of technology, such as processed EEG‐based monitoring (e.g., Bispectral Index), as a complementary tool, particularly in deeply sedated patients, alongside appropriate staff training.
4. Continuous quality improvement
  Schedule repeat audit cycles after an intervention is implemented to evaluate progress and ensure sustained performance.
5. Further research
  Investigate the clinical impact of inaccurate and incomplete RASS assessments and identify barriers to adherence using qualitative methods.

### 4.5 Strengths and Limitations

This single‐centre audit has several limitations. Generalisability may be limited due to local practices and the patient population. The use of expert assessment as a reference standard introduces subjectivity and potential confirmation bias. Documentation was used as a proxy for assessment, which might not reflect actual practice. Recall bias may also influence self‐reported training data. It is worth reiterating that RASS validity depends on direct, structured observation by trained raters [6, 18].

The specialist cardiothoracic population, including transplant and ECMO patients, likely contributed to the high proportion of deeply sedated patients (with the RASS scores at −5), limiting broader applicability. Additionally, the limited sample size reduces statistical power, and the absence of a defined sampling framework may introduce selection bias. As only univariable analyses were used, potential confounding could not be accounted for, so the findings should be interpreted with caution.

A key strength of this study is the use of audits conducted without prior notification, which likely captured more representative real‐world clinical practice and reduced observer bias. This approach aligns with evidence suggesting that announced audits may overestimate performance due to preparatory behaviour [27, 28, 29].

## 5 Conclusion

This audit identified suboptimal RASS documentation and accuracy at the Wythenshawe Hospital's Cardiothoracic Critical Care Unit, with only 33% of assessments fully documented and 25% inaccurate compared with expert assessment. While greater experience was associated with improved accuracy, confidence did not correlate with better performance, highlighting a potential gap between perceived and actual competence. These findings support the need for targeted staff education, improved documentation systems and ongoing audit cycles. Further research is warranted to determine the clinical impact of these deficiencies on patient outcomes and to evaluate strategies, including educational, workflow and objective monitoring approaches to improve sedation assessment and patient safety.

## Funding

The authors have nothing to report. The first author, however, has received a research grant from Medtronic External Research Program (ERP reference ERP‐2022‐13050).

## Disclosure

The first author acknowledges the use of Grammarly to assist with spelling and grammar correction. All AI‐assisted text was reviewed and revised by the authors to ensure accuracy and clarity of meaning.

## Ethics Statement

The survey was developed as part of the PRO‐BIS on ECMO feasibility study (https://doi.org/10.1186/ISRCTN79335747), a postgraduate research project at the University of Salford, United Kingdom, with ethical approval from the University of Salford (HSR2223‑001) on 06 December 2022 and from the UK’s Health Research Authority (REC: 22/NW/0316) on 23 March 2023.

## Consent

Participants were informed that participation was voluntary and that responses would be collected anonymously with no identifiable data retained. Participation implied verbal consent to the use of aggregated data for analysis, publication and service improvement.

## Conflicts of Interest

The authors declare no conflicts of interest.

## Data Availability

The authors confirm that the methods used are appropriate for the study design and data, and that the statistical results have been correctly carried out and interpreted. They accept responsibility for choosing suitable methods and ensuring their proper use as a condition of submission to the journal. The project data include anonymised staff assessments and summary statistics. Because the team was small and worked in a particular department, sharing the full raw data could risk identifying individual staff members. The article includes anonymised, group‐level results. A lightly edited data set and the analysis code can be requested from the corresponding author, subject to approval from Manchester University NHS Foundation Trust and in line with local information‐governance policy.
